# Extracellular Inflammasome Particles Are Released After Marathon Running and Induce Proinflammatory Effects in Endothelial Cells

**DOI:** 10.3389/fphys.2022.866938

**Published:** 2022-05-20

**Authors:** Alexander Kogel, Sven Fikenzer, Luisa Uhlmann, Lena Opitz, Jasmin M. Kneuer, Karl Georg Haeusler, Matthias Endres, Jürgen Kratzsch, Viktoria Schwarz, Christian Werner, Hermann Kalwa, Susanne Gaul, Ulrich Laufs

**Affiliations:** ^1^ Klinik und Poliklinik für Kardiologie, Universitätsklinikum Leipzig, Leipzig, Germany; ^2^ Department of Neurology, Universitätsklinikum Würzburg, Würzburg, Germany; ^3^ Department of Neurology and Center for Stroke Research Berlin, Charité—Universitätsmedizin Berlin, Berlin, Germany; ^4^ German Center for Neurodegenerative Diseases (DZNE) and German Centre for Cardiovascular Research (DZHK), Berlin, Germany; ^5^ Institute of Laboratory Medicine, Clinical Chemistry and Molecular Diagnostics, University of Leipzig, Leipzig, Germany; ^6^ Department for Internal Medicine III, Cardiology, Angiology and Intensive Care Medicine, Saarland University, Saarbrücken, Germany; ^7^ Rudolf-Boehm-Institut für Pharmakologie und Toxikologie, Medizinische Fakultät, Universität Leipzig, Leipzig, Germany

**Keywords:** marathon, NLRP3, inflammasome, cardiovascular disease, endothelial dysfunction, inflammation

## Abstract

**Objectives:** The intracellular NLRP3 inflammasome is an important regulator of sterile inflammation. Recent data suggest that inflammasome particles can be released into circulation. The effects of exercise on circulating extracellular apoptosis-associated speck-like protein (ASC) particles and their effects on endothelial cells are not known.

**Methods:** We established a flow cytometric method to quantitate extracellular ASC specks in human serum. ASC specks were quantitated in 52 marathon runners 24–72 h before, immediately after, and again 24–58 h after the run. For mechanistic characterization, NLRP3 inflammasome particles were isolated from a stable mutant NLRP3 (p.D303N)-YFP HEK cell line and used to treat primary human coronary artery endothelial cells.

**Results:** Athletes showed a significant increase in serum concentration of circulating ASC specks immediately after the marathon (+52% compared with the baseline, *p* < 0.05) and a decrease during the follow-up after 24–58 h (12% reduction compared with immediately after the run, *p* < 0.01). Confocal microscopy revealed that human endothelial cells can internalize extracellular NLRP3 inflammasome particles. After internalization, endothelial cells showed an inflammatory response with a higher expression of the cell adhesion molecule ICAM1 (6.9-fold, *p* < 0.05) and increased adhesion of monocytes (1.5-fold, *p* < 0.05).

**Conclusion:** These findings identify extracellular inflammasome particles as novel systemic mediators of cell–cell communication that are transiently increased after acute extensive exercise with a high mechanical muscular load.

## Introduction

Vascular diseases are the leading cause of mortality and disability worldwide (Roth et al., 2020). In addition to the classic risk factors such as dyslipidemia, diabetes mellitus, and arterial hypertension, vascular inflammation is recognized as a driver of endothelial dysfunction and target of therapy (Daiber et al., 2017). Clinical studies have provided promising data on risk reduction using anti-inflammatory strategies (Ridker et al., 2017; Tardif et al., 2019; Nidorf et al., 2020).

An important cytokine for the inflammatory pathogenesis of cardiovascular diseases is interleukin-1 (IL-1) (Devlin et al., 2002; Bhaskar et al., 2011; van Tassell et al., 2013). IL-1 is processed by caspase-1 after the formation and activation of the NLR family pyrin domain containing 3 (NLRP3) inflammasome complex (Geng and Libby, 1995; Martinon et al., 2002). The intracellular NLRP3 inflammasome has been identified as an important component of the pathology of several inflammatory diseases (Rheinheimer et al., 2017; Zhao et al., 2020). These diseases range from directly inflammasome-associated diseases such as the rare cryopyrin-associated periodic syndrome, the Muckle–Wells syndrome, or the familial cold autoinflammatory syndrome to metabolic disorders with high prevalence such as type 2 diabetes, atherosclerosis, obesity, and gout (Hoffman et al., 2001; Frenkel et al., 2004; Sun et al., 2017). Upon activation, NLRP3 nucleates with apoptosis-associated speck-like protein (ASC) to form an ASC speck, leading to caspase-1-mediated proteolytic activation of interleukin-1β (IL-1β) and interleukin-18 (IL-18), and the induction of inflammatory, pyroptotic cell death. Recent evidence shows that pyroptotic cells can subsequently release ASC specks into the extracellular space where they may perpetuate inflammation (Gaul et al., 2021). Extracellular ASC specks have been described in chronic obstructive pulmonary disease, septic patients, and myelodysplastic syndrome (Franklin et al., 2014; Basiorka et al., 2018; Martínez-García et al., 2019). However, the regulation and importance of circulating ASC specks are incompletely understood.

In cardiovascular diseases, the NLRP3 inflammasome is associated with endothelial dysfunction, the progression of atherosclerosis, and damage caused after reperfusion of ischemic myocardial tissue (Paramel Varghese et al., 2016; Gong et al., 2018). In the vascular wall, the NLRP3 inflammasome can be activated by pathogens such as cholesterol crystals, hypoxia, and hypercholesterolemia (Janoudi et al., 2016; Koka et al., 2017; Jiang et al., 2020). Interestingly, a genetic variant of NLRP3 with an intrinsic higher activity is associated with a higher prevalence of coronary artery disease (Schunk et al., 2021). The effects of other stressors, such as acute exercise with a high mechanical muscular load, on the inflammasome are not known.

Low physical activity and a sedentary lifestyle are risk factors for cardiovascular morbidity and mortality (Gibbs et al., 2015). The positive effects of regular physical activity on the cardiovascular system have been very well documented and are recommended as the basis of vascular prevention (Kyu et al., 2016; Hussain et al., 2018; Pelliccia et al., 2021). In contrast to regular moderate to high-intensity exercise, excessive bouts of physical activities such as marathon running have been associated with an acute upregulation of the parameters of inflammation and oxidative stress (Möhlenkamp et al., 2008; Breuckmann et al., 2009; Armstrong et al., 2015; Lear et al., 2017). Based on these observations, the “extreme exercise hypothesis” was proposed, which states that adverse cardiovascular events may occur more often following high-volume high-intensity exercise (Eijsvogels et al., 2018). The mediators for these potentially negative effects are incompletely understood.

Therefore, the aim of this study was to establish a method to quantitate ASC specks in human blood and to explore the effects of marathon running on circulating ASC specks as mediators of systemic inflammation and inflammasome activation. We hypothesized that inflammasome particles may be taken up by endothelial cells where they may induce a pro-inflammatory phenotype.

## Methods

### Flow Cytometric Analysis of Apoptosis-Associated Speck-Like Protein Specks in Human Serum

**FIGURE 1:**
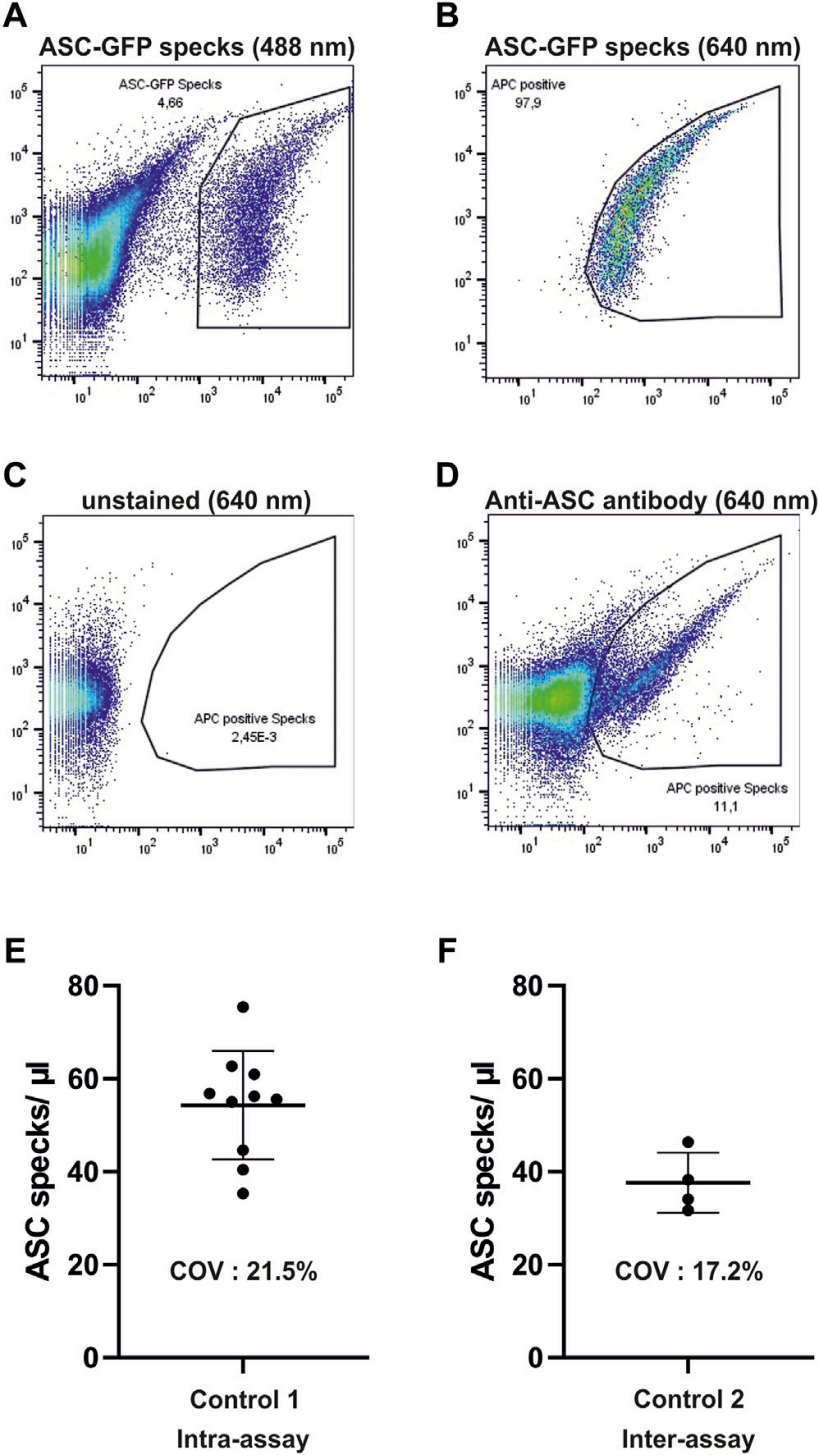
Gating strategy and validation of flow cytometric measurements of ASC specks. **(A,B)** Depicted are dot plots of isolated ASC-GFP particles which are labeled with ASC-B3-Alexa-647 antibodies in human serum in the 488 and 640-nm channels. **(C,D)** Resulting gate in the Alexa-647 channel is then used for the detection of ASC-specks in human serum after staining with AS-B3-Alexa-647 antibodies. **(E,F)** One control subject without prior exercise was measured ten times (intra-assay) and another one on four different days (inter-assay) to test for accuracy of measurements. COV, coefficient of variation.

A total of 75 µl of human serum was diluted in 225 µl phosphate-buffered saline (PBS) and centrifuged at 17,000x g for 20 min at 4°C to isolate microparticles. After centrifugation, the supernatant was discarded and the pellet was resuspended in 300 µl PBS. The samples were then stained using ASC-B3-Alexa-647 (sc-514414, Santa Cruz) for 1 h at 37°C. Afterward, 120 µl of the samples was measured using a flow cytometer BDFacsLyric (BDscience). ASC-GFP specks derived from THP1-ASC-GFP cells were labelled with an ASC-B3-Alexa-647 antibody and added to human serum to serve as a positive control for gating the samples. These were detected in the 488-nm channel of the flow cytometer (Figure 1A) and the identified population was then marked accordingly in the 640-nm channel (Figure 1B). The resulting gate was used to identify extracellular ASC specks in the serum of the human samples (Figures 1C, D). Results were then calculated as ASC specks/µl by dividing the amount of ASC specks in the gate by the volume of the probe measured accounting for dilution during the centrifugation steps. The validation of the FACS measurements was based on an established cell line—THP1-ASC-GFP (Invivogen)—for the isolation of ASC specks (Proske and Morgan, 2001). The measured particles in human serum resemble the isolated specks in granularity, size, and fluorescence intensity. Intra- and interassays on the serum of the volunteers without prior exercise yielded coefficients of variation of 21.5 and 17.2%, respectively (Figures 1E,F).

### Human Samples

Blood samples were collected from participants of the Berlin Beat of Running study, a prospective, observational, and investigator-initiated study conducted during the 38th Berlin marathon (clinicaltrials.gov, NCT01428778) (Haeusler et al., 2012). Inclusion criteria were completed for registration for the marathon run: age 35–60 years, a marathon history of at least two marathon runs within the last five years and self-reported running for at least 40 km per week. Exclusion criteria included known cardiac diseases or atrial fibrillation, known or newly detected brain disease, contraindications for (brain) magnetic resonance imaging, severe liver or kidney disease, hyperthyroidism, pregnancy, or lactation. The study protocol is in accordance with the Declaration of Helsinki and was approved by the local Ethics Committee of the Charité-Universitätsmedizin Berlin, Germany (EA4/042/ 11). All participants gave their informed consent before their inclusion in the study.

The baseline visit (t0) took place three days before the marathon. Sociodemographics, cardiovascular risk factors, and the level of physical activity were documented. Participants underwent an assessment of vital parameters (heart rate and blood pressure) and venous blood sampling (Sarstedt Monovette). Within 30 min after the end of the marathon run (t1), vital parameters were assessed and the second blood sampling was done. The third blood sampling with the aforementioned parameters and assessment of vital parameters was done up to 58 h after the race (t2) (Haeusler et al., 2012; Herm et al., 2017).

### Cell Culture

Primary human coronary artery endothelial cells (HCAEC) were purchased from Promocell (C-12221) and maintained in EGM™ Endothelial Cell Growth Media (CC-3121) and Single Quots™ Supplements (CC-4133). Human umbilical vein cells (EA.hy926) were purchased from ATCC (CRL-2922) and maintained in Dulbecco’s Modified Eagle Medium (DMEM) high glucose (Gibco). Mutant NLRP3-YFP p.D303N human embryonic kidney 293 (HEK) cells were used to isolate overactive YFP-labelled NLRP3-inflammasome oligomers as previously described (Martín-Sánchez et al., 2015). THP1-ASC-GFP cells were maintained in RPMI1640. This cell line was cultured in Dulbecco’s Modified Eagle Medium F-12 (DMEM/F-12) growth media in the presence of Geneticin G418 (2 mg/ml) to maintain mutation (Baroja-Mazo et al., 2014). Internalization of NLRP3-YFP particles in HCAEC was determined by immunofluorescence staining with anti-YFP and anti-rabbit Alexa-488 antibodies. Alexa Fluor™ 555 Phalloidin and DAPI were used for F-actin and nucleus staining, respectively.

### Isolation of Inflammasome Particles

Fluorescent p.D303N NLRP3-YFP inflammasome oligomers were isolated and purified from 1 × 10^7^ mutant NLRP3-YFP (p.D303N) HEK cells and resuspended in 100 µl non-denaturating CHAPS buffer following an established protocol (Martín-Sánchez et al., 2015). Fluorescent ASC-GFP specks were isolated and purified from THP1-ASC-GFP cells (Invivogen) and resuspended in 100 µl non-denaturating CHAPS buffer following the same protocol with the addition of a primer with LPS at 1 µg/ ml for 3 h and activation by Nigericin at 5 µM for 1 h before isolation.

### Cellular Imaging

HCAEC were treated with NLRP3-YFP particles for 24 h. Cells were fixed with 4% paraformaldehyde, permeabilized and incubated with an anti-YFP (GFP) antibody (#6556, Abcam), anti-YFP Alexa555 antibody (A-31851, Invitrogen), anti-ICAM-1 (#MA5407, Invitrogen), IgG isotype control (#RIgG, #MigG, Dianova), goat anti-mouse Cy3 (#115-165-146, Dianova), donkey anti-rabbit Alexa-488 (A-21206, Invitrogen), or goat anti-rabbit Alexa-647 secondary antibody (A-21245, Invitrogen) to analyze the internalization of extracellular NLRP3-YFP oligomers or ICAM-1 expression. DAPI, Hoechst, and F-actin Phalloidin Alexa 555/ Alexa647 were used for nucleus and F-actin staining, respectively (Gaul et al., 2021). Cells were visualized on a ZEISS Axiovert 200 M fluorescent microscope. Z-stacking (12 subsequently optical slices of 0.8 µm thickness) and 3D reconstruction were performed with AxioVision LE Rel 4.4 software. A minimum of five randomly selected fields were used. To analyze ICAM-1 expression, the evaluation of 8–10 immunofluorescence images per condition and experiment was performed by ImageJR software. First, a gray scale image was used to set a threshold from which the ICAM1 signal was considered positive. This value was applied to all images. Then, the product of the mean intensity and the area of the ICAM1 signal (integrated density) were determined. This determined value was normalized to the cell number, which was counted on the basis of the intact cell nuclei. An ImageStreamX Mk II Imaging Flow Cytometer was also used for internalization studies.

### Steroid Hormone Analysis

Steroid hormones in serum, which include cortisol, cortisone, testosterone, androstenedione, 17-hydroxyprogesterone (17-OHP), dehydroepiandrosterone sulfate (DHEAS), progesterone, and estradiol, were simultaneously quantified using liquid chromatography-tandem mass spectrometry (LC–MS/MS) as described (Bae et al., 2019).

### Monocyte Adhesion

Monocyte adhesion was assessed by calcein red-orange staining (7.5 µM) of THP1 monocytes and fluorometric analysis (Ex571/Em596) of monocytes on HCAEC. For this purpose, HCAEC were treated with extracellular NLRP3-YFP particles for 24 h and then incubated with stained THP1 monocytes for 4 h. Adherent cells were analyzed fluorometrically and normalized to untreated cells.

### Data Analysis

Analyses were performed with the Graph Pad Prism (version 7), ImageJ 1.52a, and FlowJo v10. The significance level was set at *p* < 0.05. Multiple groups were analyzed using a mixed-effect analysis and the Tukey post-hoc test. Two groups were analyzed by a two-sided Student´s *t*-test. Data are expressed as mean ± standard error of the mean (SEM) if not stated otherwise. A correlation analysis was performed using JASP 0.14.1 (JASP Team, 2020). CorelDraw2018 was used to create the artwork. Statistical outliers were identified using the ROUT method with Q = 1%.

## Results

### Marathon Running Increases Extracellular Apoptosis-Associated Speck-Like Protein Specks in Human Serum

**FIGURE 2:**
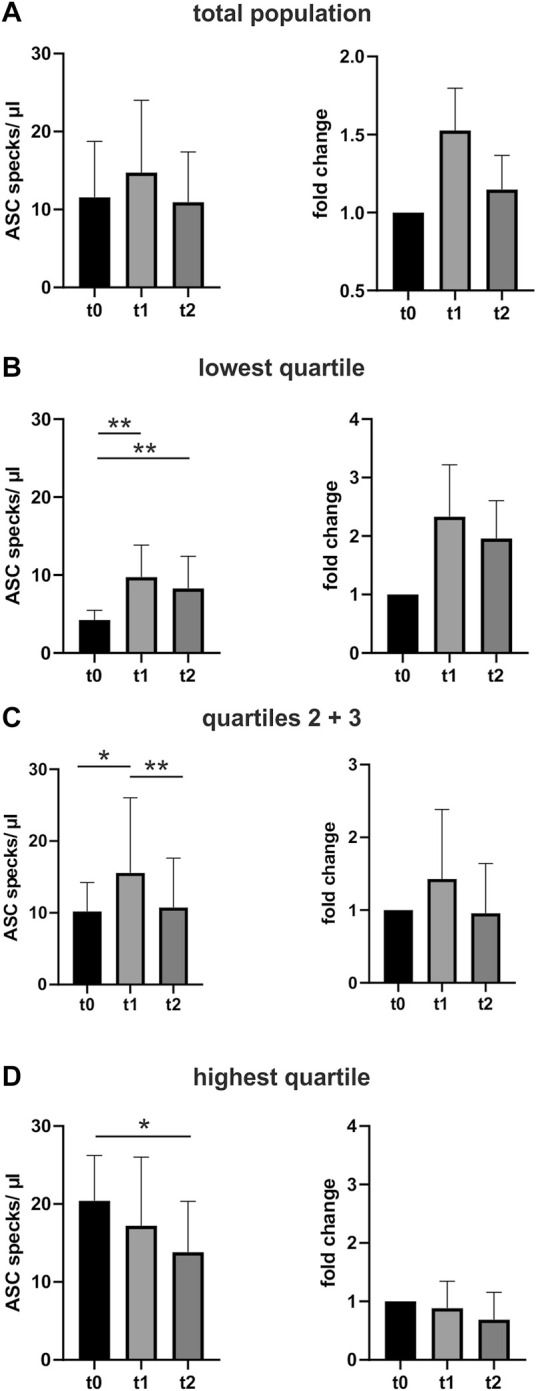
Extracellular ASC speck concentration increases after performing a marathon run and decreases again in the days after. **(A)** Extracellular ASC specks were measured using flow cytometry. Samples were collected 24–48 h before (t0) (N = 52), immediately after (t1) (N = 52), and 2–3 days after the marathon run (t2) (N = 52). Absolute values of ASC specks per microliter serum and the individual fold change to t0 are depicted. **(B–D)** Subgroup analysis of the quartile with the lowest ASC speck concentration at t0 **(B)**, the middle group **(C)**, and the quartile with the highest **(D)** ASC speck concentration at t0. Data are expressed as mean +standard deviation *p < 0.05; **p < 0.01. Mixed-effects analysis and the post-hoc Tukey test were used to analyze the data.

Baseline characteristics are shown in Table 1. Performing a marathon run resulted in a significant 1.5-fold increase in circulating ASC specks immediately after the run compared with the baseline assessment (*p* < 0.05). The absolute number of ASC specks increased from 11 ± 7 ASC specks/µl to 15 ± 9 ASC specks/µl serum. After the marathon, the concentration decreased toward baseline levels to 11 ± 6 ASC specks/µl (*p* < 0.01) (Figure 2A).

**TABLE 1:** Study population.

Parameter	Unit	Value (N = 52)
Age	years	48.8 ± 5.5
Male	No.	52
Body mass index	kg/m^2^	23.8 ± 2.0
Heart rate	bpm	62 ± 8
Systolic blood pressure	mmHg	131 ± 15
Diastolic blood pressure	mmHg	85 ± 8
Training status		
Marathon runs in total	No.	22 ± 50
Marathon runs <5 years	No.	9 ± 8
Current weekly running distance	km	66 ± 17
Regular weekly running distance	km	44 ± 14
Cardiovascular risk factors		
Hypertension	% (n)	9.6 (5)
Diabetes mellitus	% (n)	0 (0)
Hyperlipidaemia	% (n)	3.8 (2)
Current smoking	% (n)	9.6 (5)

An exploratory analysis of quartiles of baseline ASC speck concentrations at time t0 revealed that the exercise-induced regulation correlates with the baseline levels. Athletes with low baseline ASC speck levels showed a marked upregulation at t1 that persisted until t2. The middle group of athletes had a distinct increase in circulating ASC specks after the run that recovered until t2. In contrast, individuals with baseline ASC specks in the highest quartile showed no additional upregulation after the marathon. (Figures 2B–D).

### Upregulation of Extracellular Apoptosis-Associated Speck-Like Protein Specks Correlates With Stress Hormones but Not With Microparticles

**FIGURE 3:**
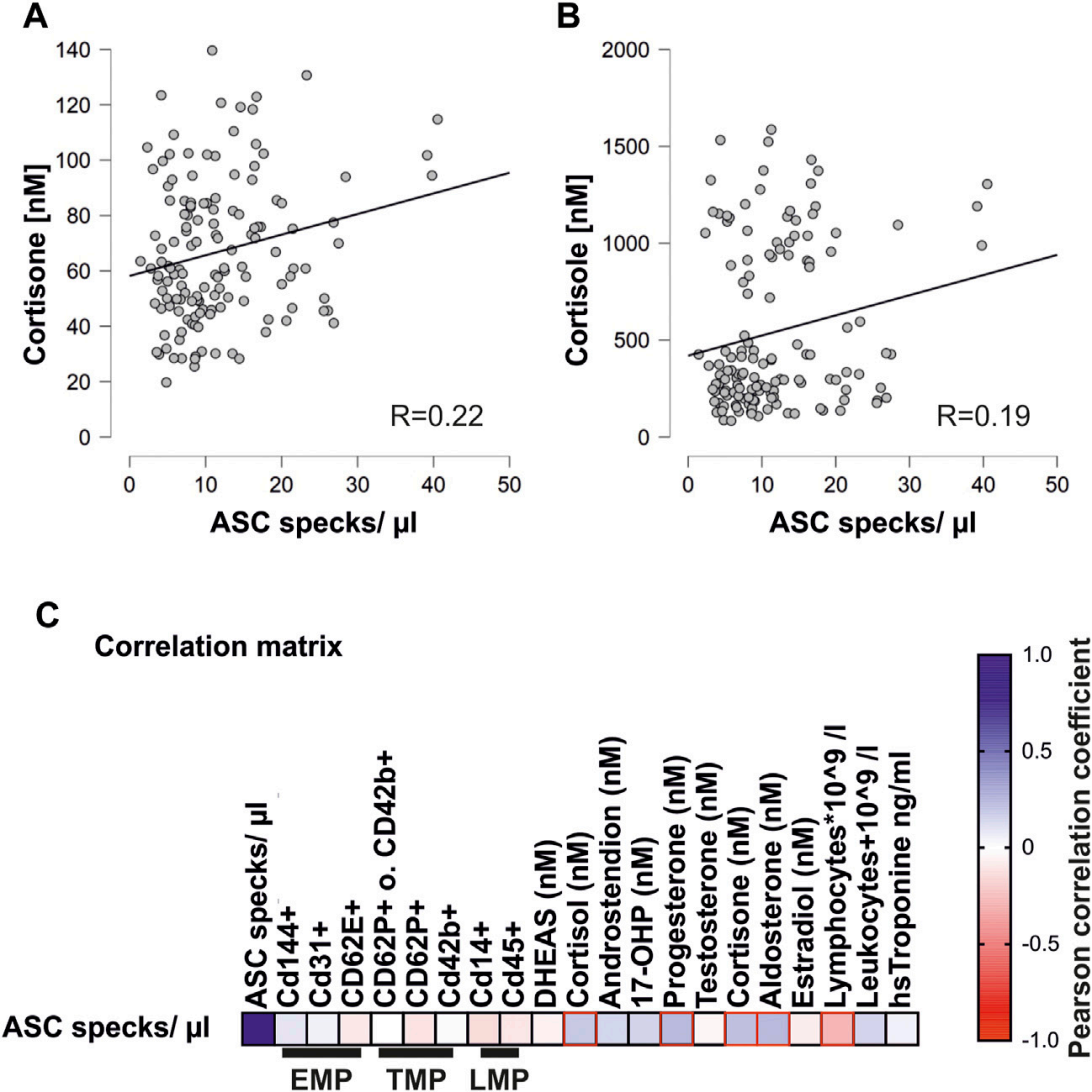
Concentrations of circulating ASC specks correlate with steroid hormones and lymphocytes **(A)** Pearson correlation matrix of extracellular ASC specks with various endothelial (EMP), platelet (TMP), and leukocyte (LMP) microparticles and steroid hormones, lymphocytes, leukocytes, and high-sensitivity troponin. Significant correlations (*p* < 0.05) are marked in red. **(B,1C)** Exemplary scatter plots and linear regression of correlations of extracellular ASC specks with cortisone and cortisol. The Pearson correlation coefficient was used to analyze the data.

Marathon running induces the upregulation of stress hormones and cellular microparticles (Bae et al., 2019; Schwarz et al., 2018). The ASC specks showed a significant positive correlation with stress hormones such as cortisone (R = 0.22), cortisol (R = 0.19), aldosterone (R = 0.24), and progesterone (R = 0.24) (Figures 3A,B) (Bae et al., 2019).

In addition, there was a significant negative correlation with the number of circulating lymphocytes that are known to decrease after severe endurance exercise (R = 0.27) (Santos et al., 2013).

In contrast to the stress hormones, there was no correlation of the concentration of extracellular ASC specks with circulating microparticles. These included endothelial, platelet, and leukocyte microparticles (Figure 3C) (Schwarz et al., 2018). The lack of correlation between the ASC specks and the different types of microparticles is an indicator of the specificity of the circulating ASC particles as released by inflammasome activation rather than more general cell damage.

### Human Coronary Endothelial Cells Internalize Extracellular NLRP3 Inflammasome Particles

**FIGURE 4:**
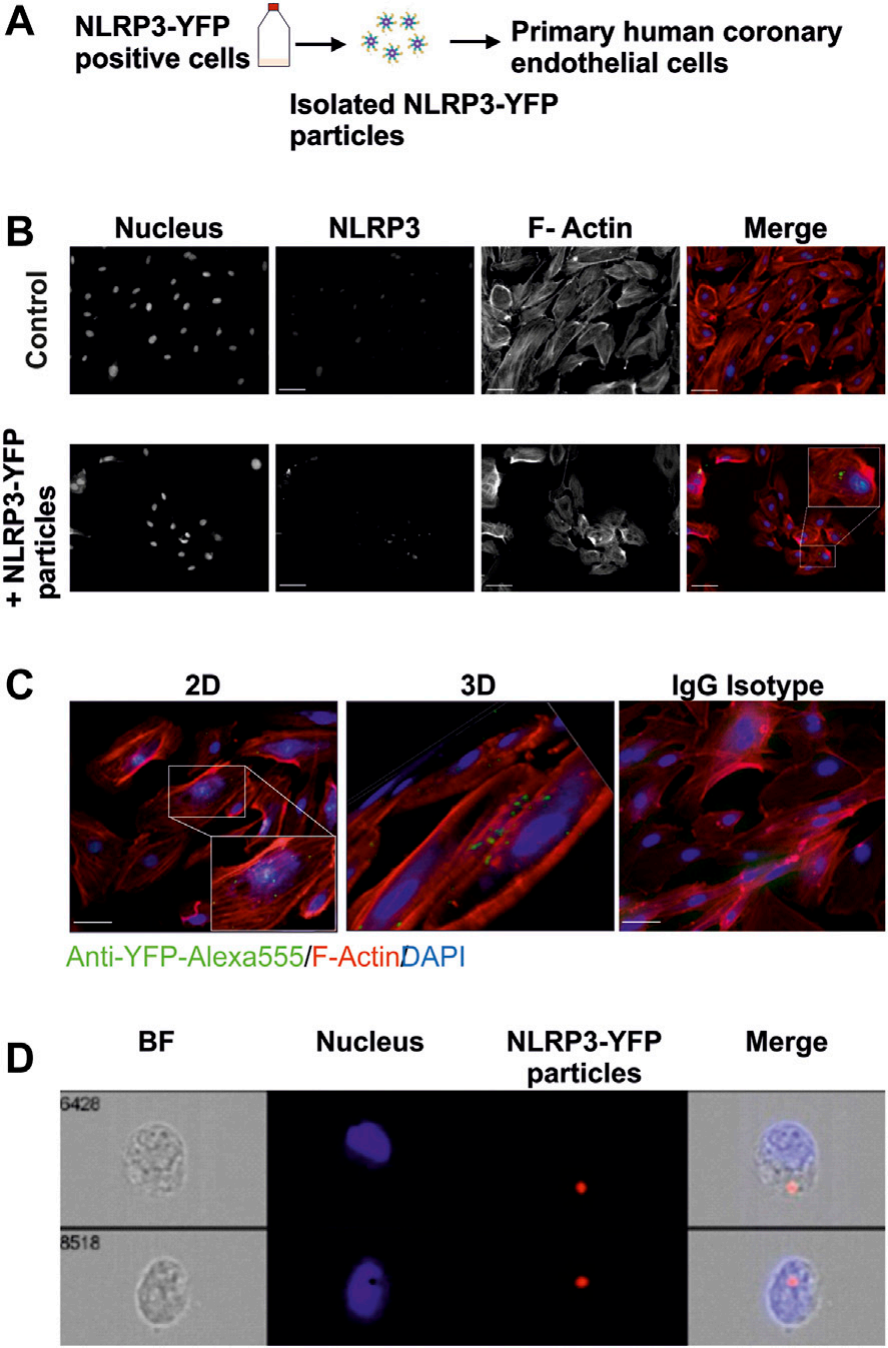
Extracellular NLRP3-YFP particles are internalized by primary human coronary artery endothelial cells. **(A)** Schematic overview. Mutant NLRP3 (p.D303N)-YFP HEK cells are used to isolate oligomeric NLRP3-YFP inflammasome particles and treat primary human coronary artery endothelial cells. **(B)** Internalization of extracellular NLRP3-YFP particles in primary human coronary artery endothelial cells (HCAEC) after 4 h of incubation and determined by immunofluorescent staining (N = 3) with a primary fluorescently labeled anti-YFP-Alexa555 antibody (green) (scale bar: 50 µm). Alexa Fluor™ 647 Phalloidin (red) and DAPI (blue) were used for F-actin and nucleus staining, respectively. Z-stacks with xz and yz focal planes showing internalized NLRP3-YFP inflammasome particle. **(C)** Z-stacks were used for 3D reconstruction (scale bar: 50 µm). **(D)** Two representative ImageStream^®^ analyses of HCAEC including bright field (BF), DAPI (Nucleus), 647/Cy5 Channel (Ex. NLRP3), and a merge of an internalized extracellular NLRP3-YFP inflammasome are shown (scale bar: 10 µm).

To test whether extracellular NLRP3-YFP inflammasome particles are taken up by human endothelial cells, immunofluorescent staining of internalized YFP-tagged NLRP3 inflammasomes and cell organelle staining were performed (Figure 4A). Internalization of extracellular NLRP3-YFP inflammasome particles into HCAEC was analyzed by nucleus (DAPI) and cytoskeleton (F-Actin Phalloidin) staining and the detection of intracellular YFP-positive signal. The intracellular NLRP3-YFP particles are located in the region around the nucleus (Figures 4B–D).

### Extracellular NLRP3-YFP Particles Induce a Pro-Inflammatory Response in Human Coronary Endothelial Cells.

**FIGURE 5:**
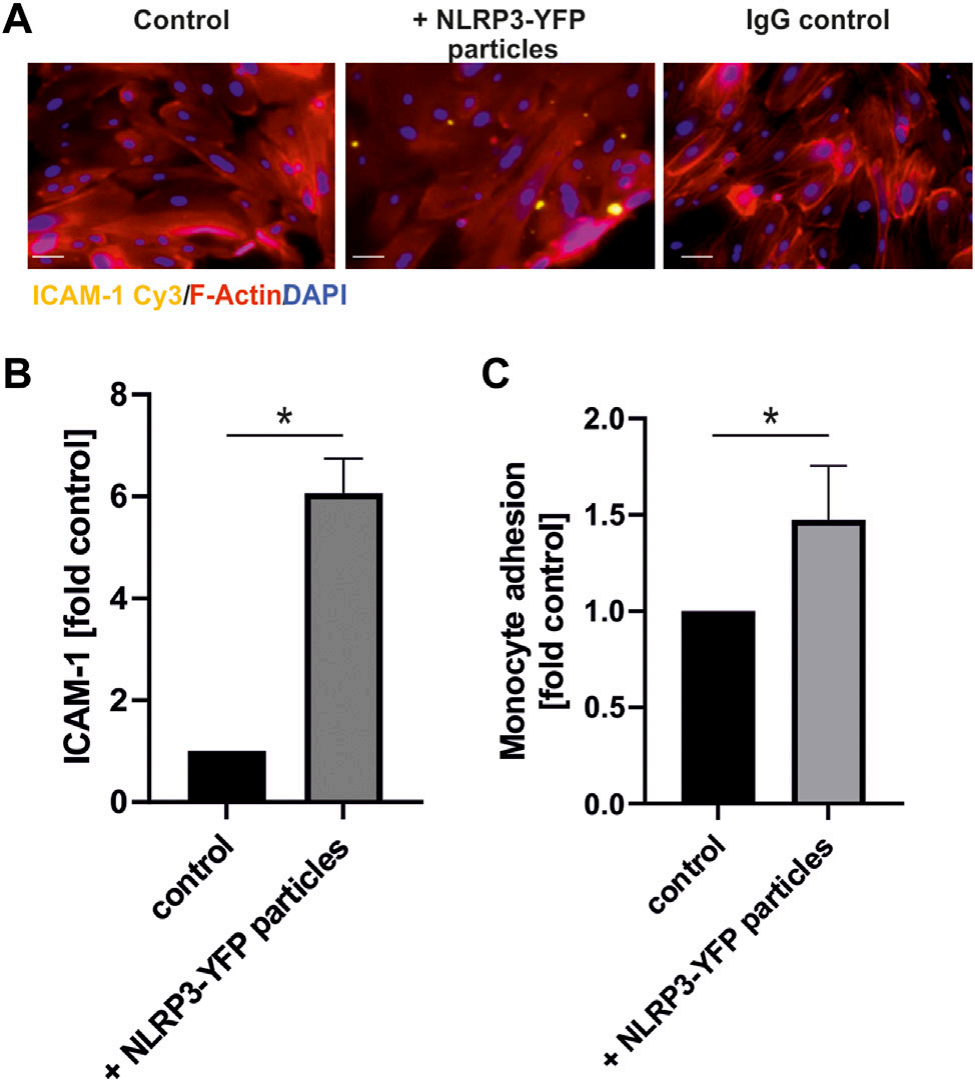
Treatment with extracellular NLRP3 inflammasome particles induces inflammation in endothelial cells. **(A)** Immunofluorescent analysis of ICAM-1 protein expression on coronary artery endothelial cells after 24 h incubation with NLRP3-YFP particles (3 NLRP3-YFP particles/cell) and stained with anti-ICAM1 Cy3 (scale bar: 50 µm). **(B)** Quantitative analysis of ICAM-1 fluorescence (N = 4). **(C)** Calcein-stained THP-1 monocyte adhesion on endothelial cells after incubation with NLRP3-YFP particles for 4 h measured by a fluorometric analysis and normalized to untreated cells in the culture medium (control). Two-sided unpaired *t*-test was used to analyze the data.

Treatment of HCAEC with NLRP3-YFP particles (3 NLRP3-YFP particles/cell for 24 h) increased the expression of the cell adhesion molecule ICAM-1 measured by immunofluorescent staining by 6.9-fold (*p* < 0.05) (Figures 5A,B).

Additionally, monocyte adhesion on endothelial cells was increased by the extracellular NLRP3-YFP particles by 1.4-fold (*p* < 0.05) (Figure 5C).

## Discussion

The main findings of the study are the increase of circulating extracellular ASC specks in human serum immediately after a marathon run, the uptake of extracellular inflammasome particles by endothelial cells, and the induction of endothelial cell inflammation. Marathon running induces a marked but transient increase of circulating ASC specks. Mechanistically, the data reveal that cell-free extracellular inflammasome particles can be internalized by human endothelial cells and promote an inflammatory response characterized by the upregulation of ICAM-1 and increased monocyte adhesion. The data show that extracellular ASC specks represent markers of vascular inflammation.

### Extracellular Apoptosis-Associated Speck-Like Protein Specks After a Marathon

Extracellular inflammasome particles are generated and released when cells undergo pyroptosis. The number of these particles in the blood reflects pyroptotic cell damage. The upregulation of the ASC specks showed an inverse correlation with baseline levels, the lower the baseline, the higher the marathon-induced upregulation. These data suggest a maximum saturation of the exercise-induced number of circulating inflammasome particles. Further studies are needed to address whether lower baseline ASC speck levels relate to individual predisposition or the amount of exercise performed by different athletes in the days before the marathon run. The variance of baseline ASC speck levels may be attributed to different lifestyles, pre-existing conditions or nutrition, and the microbiome in the gut (Youm et al., 2015; Ding et al., 2019). The number of ASC specks in the serum does not correlate with endothelial, platelet, and leukocyte microparticles as a correlate of cell damage. This observation supports the importance of ASC specks as an independent factor. The mechanical damage of myocytes induces the release of typical inflammasome activators such as extracellular ATP or reactive oxygen species which then results in a cascade of inflammasome activation not only in the surrounding muscles but also vascular cells (Bailey et al., 2004; Bauernfeind et al., 2011; Heid et al., 2013; Panicucci et al., 2020). The cell damage of myocytes is documented by the release of typical proteins, miRNAs, and the clinical presentation of muscle soreness after excessive bouts of endurance exercise (Uhlemann et al., 2014; Bernat-Adell et al., 2021). Therefore, the increased ASC specks after marathon running may originate from skeletal muscle cells that are subjected to high physical strain (Pincheira et al., 2021).

An increase of the circulation of ASC in plasma of cyclists post-exercise using Western blot has already been shown; we see similar results in marathon runners and also a similar variance in our baseline values (Nieman et al., 2020). It is also known that exercise results in an increased expression of NLRP3 indicating that it is the relevant sensor protein to these ASC oligomers (Li et al., 2015; Khakroo Abkenar et al., 2019).

The increase in the circulation of ASC particles after the marathon is in agreement with the observation of upregulation of circulating ASC in the plasma of cyclists (Nieman et al., 2020) and an increased expression of NLRP3 post-exercise (Li et al., 2015; Khakroo Abkenar et al., 2019). We used the flow cytometry method to measure extracellular ASC specks. Compared with Western blot (Nieman et al., 2020), flow cytometry has the advantage that the particles are defined more precisely by spiking experiments and that flow cytometry allows a high turnover with highly standardized, parallel, and quantitative measurements.

### Effect of Extracellular Inflammasome Particles on Cardiovascular Cells

The study shows for the first time that endothelial cells internalize inflammasome particles leading to inflammation exemplified by the increase of ICAM-1 expression and the increase of monocyte adhesion. These data identify extracellular inflammasome particles as a mechanism that mediates inflammation from the site of damaged tissue to the endothelium. Previous findings of our group demonstrated that extracellular NLRP3 particles are also ingested by human coronary artery smooth muscle cells and macrophages where they induce pro-inflammatory and atherogenic signaling by increasing the extracellular matrix production, promoting the secretion of pro-atherogenic and inflammatory cytokines and cell migration (Gaul et al., 2021).

Extensive exercise with a high mechanical muscular load can mediate systemic inflammation via the release of extracellular inflammasome complexes. We and others propose that these pyroptosis-derived NLRP3 inflammasome particles exert pro-atherogenic effects (Gaul et al., 2021; Sun et al., 2021).

### Inflammation and Extreme Exercise Hypothesis

According to the “extreme exercise hypothesis,” long-term excessive training is associated with an increase in the overall mortality (Eijsvogels et al., 2018). Marathon runners show a higher prevalence of coronary artery calcification scores ≥100 Agatston units compared with the general population (36 vs. 22%) indicating coronary artery atherosclerosis (Möhlenkamp et al., 2008). Champion athletes have more coronary calcifications compared with sedentary men (44.3 vs 22.2%) (Merghani et al., 2017). One critical factor for cardiovascular risk is endothelial cell physiology (Souilhol et al., 2018). Therefore, the internalization of circulating pro-inflammatory inflammasome particles into endothelial cells and smooth muscle cells is likely relevant in the progression of cardiovascular diseases.

Interestingly, athletes have more stable calcified plaque phenotypes than controls and fewer mixed plaques (Merghani et al., 2017). This could indicate different underlying pathomechanisms of atherosclerosis in athletes and the general population. The role of inflammation and immune cells in the development of various plaque types is incompletely understood. Circulating inflammasome particles may be an important component in this process by increasing monocyte adhesion to endothelial cells (Waring et al., 2021).

### Limitations

There are limitations to our study. Our study population included only non-professional athletes who completed high-level endurance exercises, so transferring the data to other intensities or forms, such as strength exercise, is not possible. Nevertheless, marathon runs at competitive levels are typical for extremely intensive endurance exercise and are practiced worldwide by an increasing number of individuals. The studied population is large for a marathon study but addresses a selected and homogenous population. Therefore, the results may not apply to the general population. Measurements were performed in human serum. Other tissues are not available. Because the serum was stored for several years, the proteins may have been degraded (Cuhadar et al., 2013). Since we are only measuring one relevant protein and this should be changed in the same way in all samples, this should not change the dynamic of the ASC specks. This is also the reason why cytokines could not be measured in this study, since cytokines are extremely dependent on the storage time and conditions (Jager et al., 2009).

## Conclusion

In conclusion, the data show the release of inflammasome particles in response to extensive exercise with high mechanical muscular load, endurance exercise, and their internalization in endothelial cells as a novel mechanism causing vascular inflammation.

## Data Availability

The raw data supporting the conclusion of this article will be made available by the authors, without undue reservation.
